# Laser Desorption/Ionization on Au@TiO_2_ Core@Shell Nanostars for Mass Spectrometric Analysis of Small Molecules

**DOI:** 10.3390/nano14231946

**Published:** 2024-12-04

**Authors:** Hye-Sun Cho, Jueun Koh, Gyeonghye Yim, Hongje Jang, Young-Kwan Kim

**Affiliations:** 1 Department of Chemistry, Dongguk University-Seoul Campus, 30 Pildong-ro 1-gil, Jung-gu, Seoul 04620, Republic of Korea; sub06093@dgu.ac.kr; 2 Department of Chemistry, Kwangwoon University, 20 Gwangwoon-ro, Nowon-gu, Seoul 01897, Republic of Korea; gojooeun@naver.com (J.K.); khyim0122@naver.com (G.Y.)

**Keywords:** laser desorption/ionization, nanocomposite, TiO_2_, Au nanoparticle, mass spectrometry

## Abstract

The core@shell nanostars composed of star-like Au nanocores with TiO_2_ shells (Au@TiO_2_ NSs) are synthesized in a one-pot reaction without any reducing or surface-controlling agents. The Au@TiO_2_ NSs exhibit strong absorption in the UV region based on the interaction between the Au nanocore and the TiO_2_ shell, and this optochemical property leads to the efficient laser desorption/ionization time-of-flight mass spectrometry (LDI-TOF-MS) analysis of small molecules with low background interference and high reproducible mass signals compared with spherical Au nanoparticles (NPs). The limit of detection and dynamic range values of various analytes also improved with Au@TiO_2_ NSs compared with those obtained with spherical Au NPs. Our findings successfully demonstrate that Au@TiO_2_ NSs are a promising matrix for the LDI-TOF-MS analysis of various small molecules as well as synthetic polymers.

## 1. Introduction

Matrix-assisted laser desorption/ionization time-of-flight mass spectrometry (MALDI-TOF-MS) is one of the most powerful analytical techniques for the mass spectrometric analysis of large molecules such as nucleic acids, proteins, and synthetic polymers owing to its high resolution, sensitivity, simple and rapid analytical process, and compatibility to high-throughput analysis [1]. Owing to those characteristics, there have been huge demands to harness this analytical technique for the mass spectrometric analysis of small molecules, but the direct analytical application of MALDI-TOF-MS to small molecules has been mainly restricted by the matrix interference in the low-mass region (<500 Da) derived from the undesired side reactions of organic matrices under energetic conditions [2]. To address this critical issue, many efforts have been devoted to the development of analytical strategies for organic matrix-free laser desorption/ionization time-of-flight mass spectrometry (LDI-TOF-MS) using various nanomaterials including carbon nanofibers [2]; two-dimensional nanomaterials such as graphene and graphene oxide (GO) [3], transition metal dichalcogenide (TMDC) [4], and Mxene [5]; metal–organic frameworks (MOFs) [6]; metallic nanoparticles (NPs) [7]; and semiconductor NPs [8].

Those nanomaterials possess their own unique advantages and disadvantages based on their physicochemical properties. Two-dimensional nanomaterials such as graphene, TMDC, and MXene derivatives provide a high laser absorption capacity, thermal stability, and electrical and thermal conductivity [3,4,5], but they are generally unstable in aqueous conditions and require complicated and expensive synthetic procedures [9]. MOFs exhibit promise owing to their high laser absorption capacity, porous structure, and large surface area [6], but they also still need a complicated synthetic procedure and surface modification for dispersion in aqueous media [10]. Although metal and semiconductor NPs have been most extensively investigated owing to their high melting temperature, laser absorption capacity, and simple synthesis [7,8], they still suffer from the inevitable necessity of surface modification for stable dispersion in biological media and relatively low laser desorption/ionization (LDI) efficiency [11,12].

A promising approach to develop a highly efficient nanomaterial as a matrix for LDI-TOF-MS analysis involves the design and synthesis of nanohybrid materials, in which each component exhibits distinctive physicochemical properties essential for optimal laser desorption/ionization including key properties for high laser absorption cross-section, photochemical exciton generation, photothermal conversion, and thermal conductivity and stability. Recently, plasmonic core@shell NPs and their arrays have demonstrated significant potential in early cancer diagnosis and metabolomics based on their high LDI efficiency through the rough surface and facilitated the production of hot electrons with numerous nanogaps [13,14]. However, the synthesis and assembly of these core@shell NPs are cumbersome and non-reproducible processes, requiring multiple synthesis steps to achieve structural hierarchy. In addition, the reducing agents and nanostructural controlling compounds, which should be introduced selectively depending on the utilized elemental species, and the decrease in yield and colloidal stability due to repeated purification are also considered factors to be improved. Recently, new synthetic routes through electromagnetic radiation and the like have been continuously reported to overcome these problems, but it is also important to solve them by applying simpler chemical reactions [15,16].

**Figure 1:**
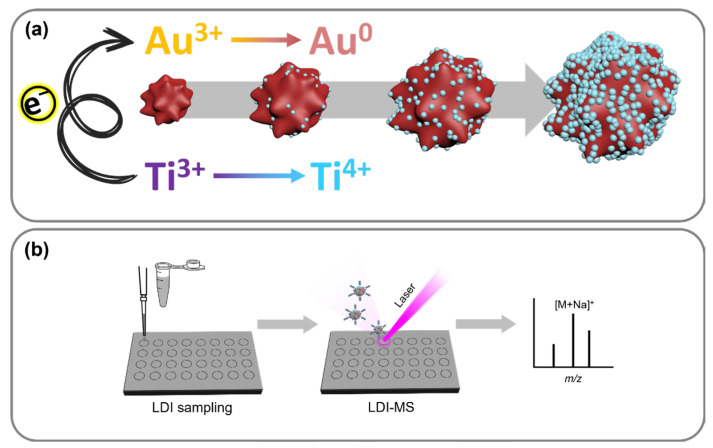
Schematic illustration of (**a**) synthesis of Au@TiO_2_ NSs and (**b**) their application to LDI-TOF-MS analysis.

In this study, the core@shell nanostars (NSs) composed of star-like Au cores with TiO_2_ shells were synthesized in a one-pot–one-step reaction without any reducing agents or surface-controlling molecules. The preparation of Au@TiO_2_ NSs consisted of an inter-cation redox reaction, which is a spontaneous electron transfer between two transition metal ions, reducible Au(IV) and Ti(III) with a multivalent potential (Figure 1a). The formation of star-like Au nanocores followed by a surface TiO_2_ coating growth is a facile and robust alternative to the synthesis of conventional core@shell NPs that omits the stepwise process altogether [17]. The manufactured Au@TiO_2_ NSs exhibit excellent absorption in the ultraviolet (UV)–visible (Vis)–near infrared (NIR) overall region from the anisotropic Au core, showing promise for a variety of photochemical applications. In addition to the intrinsic feature of the Au core, the outermost TiO_2_ shell can receive excited electrons from the core to act as a photocatalyst. Since no additives other than the metal cations (Au and Ti) are included in the synthesis, the surface of Au@TiO_2_ NSs is mostly exposed without coverage and can be utilized for adsorption, analysis, and photochemical reactions without any negative interferences.

The synthesized Au@TiO_2_ NSs were harnessed as a matrix for the LDI-TOF-MS analysis of various small molecules such as saccharides, amino acids, peptides, organic pollutants, and fatty acids as well as synthetic polymers (Figure 1b). All the tested analytes were successfully detected without severe background signals, and their mass spectrometric signals were correlated with their concentration for quantitative analysis. The LDI efficiency of the Au@TiO_2_ NSs was also compared using a model thermometer molecule, benzyl pyridinium salt, with other conventional nanomaterials including Ti_2_C MXene, GO, MoS_2_ sheets, and Au NPs, which are representatives of MXenes, graphene derivatives, TMDCs, and metallic NPs, respectively. Especially, the Au NPs were considered as an important control to clearly confirm the effect of the core@shell structure on LDI-TOF-MS analysis; therefore, the limit of detection (LOD) and dynamic range of Au@TiO_2_ and Au NPs were parallelly compared under identical conditions. As a result, it was clearly demonstrated that Au@TiO_2_ NSs are more efficient matrices than Au NPs for the LDI-TOF-MS analysis of various small molecules. Our study will provide a simple and powerful way to develop an efficient LDI-TOF-MS analytical platform for small molecules.

## 2. Materials and Methods

### 2.1. Materials

Gold(III) chloride hydrate, titanium(III) chloride (20% *w*/*v* solution in 2 M HCl), enkephalin, and bradykinin were purchased from Sigma-Aldrich (St. Louis, MO, USA). Glucose, mannitol, sucrose, cellobiose, Glu, His, Phe, myristic acid, arachidic acid, polyethylene glycol (PEG) 400, PEG 1000, PEG 2000, and benzo[a]pyrene (B[a]P) were purchased from Alfa Aesar (Ward Hill, MA, USA). Stearic acid was purchased from TCI (Tokyo, Japan). Palmitic acid was purchased from Acros Organics (Waltham, MA, USA). Asn, ethanol, tetrahydrofuran (THF), and sodium citrate dihydrate were purchased from Daejung Chemicals (Siheung-si, Republic of Korea).

### 2.2. Synthesis of Au@TiO_2_ via ICR Reaction

To a 50 mL transparent borosilicate glass vial, 40 mL of DI water was added and heated to 60 °C in a water bath. To the vial, 105 μL of 254 mM AuCl^4−^ aqueous solution was added; then, 618 μL of TiCl_3_ 20% *w*/*v* solution in 2 M HCl was directly injected under continuous stirring at 200 rpm. After 10 min of incubation, the product was purified by centrifugation at 9000 rpm for 10 min and washed with DI water at least 3 times.

### 2.3. Synthesis of Au NP

In a 100 mL lab bottle, 62.5 μL of 200 mM AuCl^4−^ aqueous solution was added to 50 mL of DI water and stirred. The bottle was heated until it boiled. When the water started boiling, 250 μL of 34 mM sodium citrate aqueous solution was added. After 10 min of incubation, the product was cooled down to room temperature under continuous stirring.

### 2.4. Characterization

The morphology and size of the nanoparticles were characterized by energy-filtering TEM (LIBRA 120, Carl Zeiss, Oberkochen, Germany). HR-TEM images and EDS mapping were achieved by using a Cs-corrected scanning TEM (JEM-ARM200F, JEOL, Tokyo, Japan). X-ray analyses were achieved by X-ray photoelectron spectroscopy (XPS) (K-Alpha+, ThermoFisher Scientific, Waltham, MA, USA) and an X-ray diffraction (XRD) system (SmartLab, Rigaku, Tokyo, Japan). A UV-Vis spectrophotometer (Lambda-465, PerkinElmer, Waltham, MA, USA) was applied to obtain the UV-Vis-NIR spectrum. The DLS and the zeta potential were characterized by a Zetasizer Nano ZS (Malvern, UK).

### 2.5. LDI-TOF-MS Analysis

The LDI-TOF-MS analysis was conducted using an IDSys (ASTA, Suwon-si, Republic of Korea), equipped with a 343 nm Nb:YAG (1 kHz) laser on a MALDI target plate. The laser intensity was maintained at 60%, with an acceleration voltage of 18 kV in the positive ionization mode. Each LDI-TOF-MS spectrum was produced by averaging 100 individual spectra, using ASTA’s custom software v17.3.4. Briefly, 1 µL of 1 mM small molecule solutions were spotted onto the target plate, followed by the addition of 1 µL of the synthesized Au@TiO_2_ nanostars and Au nanoparticles. The droplets were mixed by pipetting, allowed to dry under ambient conditions, and then analyzed. Only signals with an S/N ratio of at least 5 were considered valid mass peaks.

## 3. Results and Discussion

The formation of a plasmonic nanostructure with excellent light absorption over a wide wavelength range and the compartmentalized growth of a transition metal oxide nanocoating that will function as photocatalysts could be easily achieved through an ICR reaction. Basically, the ICR reaction consists of an electron transfer between a metal cation with a high reduction potential and a multivalent transition metal cation with a relatively low reduction potential that can act simultaneously as a reducing agent and as a precursor for the photocatalyst layer formation. Ti^3+^ was selected as the reactant for the introduction of Ti oxide (Ti^4+^|Ti^3+^ = −0.092 V vs. standard hydrogen electrode (SHE)), which has a wide bandgap (>3.2 eV) and has a photocatalytic property under UV irradiation. As a counter-reactant, the noble metal element Au (Au^3+^|Au = 1.41 V vs. SHE) was applied, which is a typical plasmonic NP in the Vis and NIR regions and has extremely high chemical stability.

The mixing of the two cations (Ti^3+^ and Au^3+^) in a polar solvent resulted in the growth of Au@TiO_2_ NSs within minutes of reaction time without the addition of any surfactants, reducing agents, polymers, or surface-stabilizing compounds. From the point of view of utilization as an analytical medium, this green synthesis, which does not have any kind of “chemical contamination” due to surface organic matter for colloidal stability, becomes one of the biggest advantages of Au@TiO_2_ NSs as a matrix for LDI-TOF-MS analysis because it can enhance the interaction with analytes and thus improve energy transfer efficiency and minimize interference from the undesired LDI of surface-adsorbed organic molecules.

**Figure 2:**
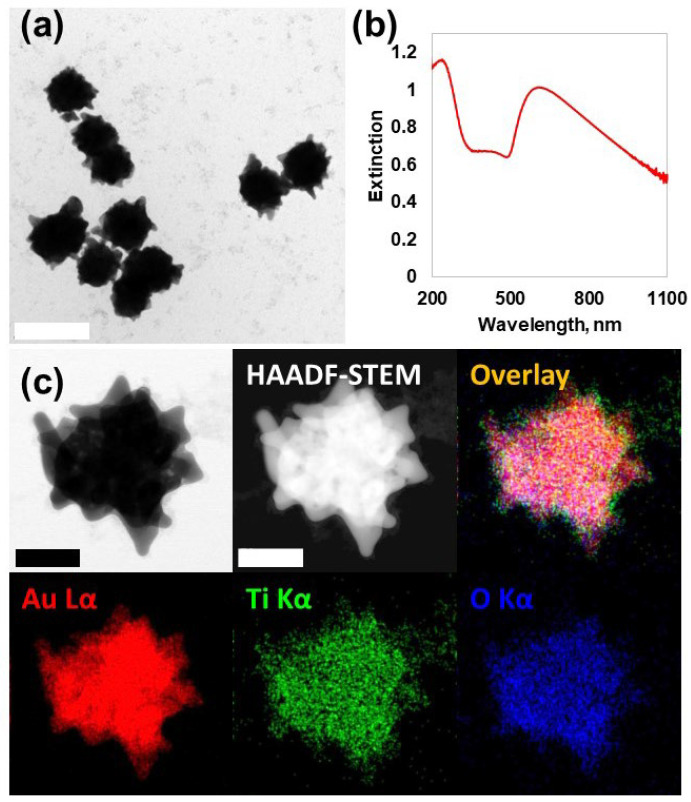
Characterization of Au@TiO_2_ NSs. (**a**) TEM images exhibited sharply protruding star-like morphology. The scale bar is 200 nm. (**b**) UV-Vis-NIR spectrum showed a strong absorption pattern in the UV and NIR regions. (**c**) HAADF-STEM/EDS mapping of single Au@TiO_2_ NSs. The scale bar is 50 nm.

According to the transmission electron microscopy (TEM) images, the formation of homogeneous particles of 146 ± 11.4 nm diameter (counting# = 50) with sharp protrusions was clearly identified (Figure 2a). The formation of star-like nanoparticle structures can be explained by a growth mechanism transitioning from kinetic to thermodynamic control during cationic redox reactions. In the initial stages, the anisotropic growth is predominantly driven by kinetic factors, favoring the rapid formation of protrusions or tips. As the reaction progresses, the system transitions to a thermodynamic regime, stabilizing the overall structure and morphology through equilibrium-driven processes. This dual-phase growth mechanism effectively accounts for the unique star-like morphology observed in the NPs [17]. The hydrodynamic diameter of water-dispersed Au@TiO_2_ NSs was calculated as 214.2 nm with 41.8 nm of standard deviation (Figure S1). The significantly large DLS distribution compared with the TEM image was interpreted to be due to the hydrophilic TiO_2_ coating on the surface in addition to the anisotropic morphology. The UV-Vis-NIR spectrum represented a strong sub-365 nm UV peak and Vis-NIR absorption band originating from the TiO_2_ shell and anisotropic star-like Au nanocore, respectively (Figure 2b) [18,19,20]. Au NPs, which were synthesized via the Turkevich–Frens method, were used as controls [21]. The TEM images showed that these particles had a uniform spherical shape with sizes ranging from 50 to 60 nm (Figure S2a), which is consistent with the wavelengths observed in their UV-Vis absorption bands (Figure S2b) [22].

The thin TiO_2_ shells were not clearly discernible in normal TEM images, so high-angle annular dark-field scanning transmission electron microscopy (HAADF-STEM) with energy-dispersive spectroscopy (EDS) characterization was performed to confirm the specific composition and distribution. The core domain, which showed a sharp contrast in HAADF-STEM caused by dense atomic nuclei (Z = 79), was Au Lα, while the presence of a Ti oxide compositional shell domain in the peripheral region was demonstrated from the co-localization of Ti Kα and O Kα signals (Figure 2c).

**Figure 3:**
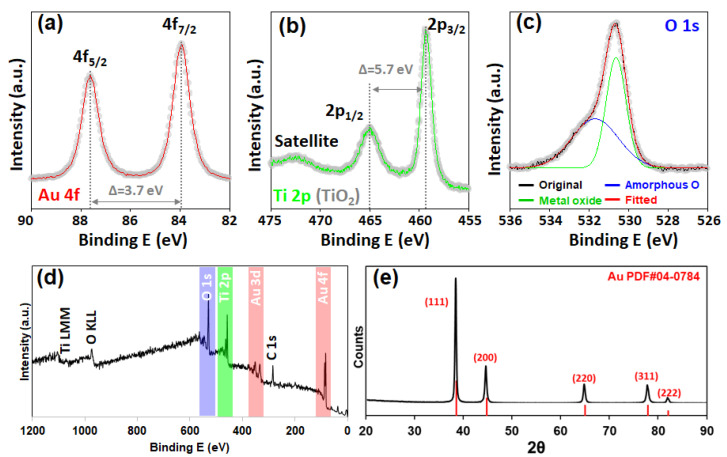
X-ray characterization of Au@TiO_2_. XPS spectra for (**a**) Au 4f, (**b**) Ti 2p, and (**c**) O 1s. (**d**) Wide-scan survey for Au@TiO_2_ film. (**e**) XRD pattern of Au@TiO_2_ exhibited a well-correlated simulated Au pattern.

More specific information on the composition and crystallinity was identified by X-ray spectroscopy. A clear Au 4f peak was identified from the XPS analysis. The two asymmetrically shaped peaks (83.9 eV and 87.6 eV for 4f_7/2_ and 4f_5/2_, respectively) with a well-separated spin–orbit component of 3.7 eV demonstrate the presence of metallic Au^0^ (Figure 3a). For the Ti 2p binding energy, the TiO_2_ composition was confirmed from the charge-transfer satellite peak around 472 eV with two distinct peaks at 459.3 eV (2p_3/2_) and 465.0 eV (2p_1/2_) with a 5.7 eV spin–orbit component (Figure 3b). The deconvoluted O 1s spectrum was identified as a metal oxide peak at 530.6 eV and an amorphous O peak at 531.7 eV (Figure 3c). In the wide-scan XPS survey, Ti 2p and O 1s peaks along with sharp Au 4f and 3d peaks were identified, proving the successful formation of Au@TiO_2_ NSs via the ICR reaction (Figure 3d) [23]. XRD analysis of the Au@TiO_2_ NSs revealed a significant Au pattern. The observed peaks at 38.2° (111), 44.4° (200), 64.6° (220), 77.5° (311), and 81.7° (222) are perfectly correlated with PDF#04-0784 (Figure 3e) [24]. All these characterization results indicated that the Au@TiO_2_ NSs were successfully synthesized under our experimental conditions.

**Figure 4:**
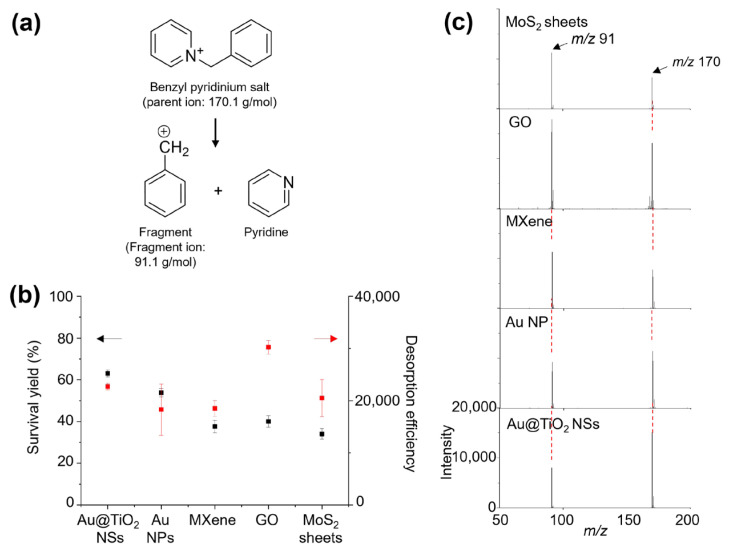
(**a**) The fragmentation reaction of BP under LDI-MS analysis. (**b**) Survival yield (black square mark and arrow), desorption efficiency (red square mark and arrow), and (**c**) LDI-TOF-MS spectra of 1 nmol BP obtained with Au@TiO_2_ NSs, Au NP, MXene, GO, and MoS_2_ sheets.

Before evaluating Au@TiO_2_ NSs as an LDI-TOF-MS platform, the optimal concentration of the Au@TiO_2_ NSs was determined by testing various concentrations of Au@TiO_2_ NSs (10, 5.0, 2.5, 1.0, 0.5, 0.25, and 0.10 eq) for the LDI-TOF-MS analysis of 1 nmol of leu-enkephalin, glucose, and histidine. At the concentration of 2.5 eq, the analytes exhibited the highest signal intensity with a clear background (Figure S3). Therefore, all the subsequent LDI-TOF-MS analyses were conducted using 2.5 eq Au@TiO_2_ NSs. Benzylpyridinium salt (BP) was then utilized to obtain the desorption efficiency (DE) and survival yield (SY) of the Au@TiO_2_ NSs. The DE and SY values are crucial parameters for estimating the LDI efficiency and soft ionization property of various nanomaterials, and BP is widely used to determine the DE and SY [25]. The DE and SY values are calculated as the total ion intensity of BP and its fragment ions and as the relative intensity of BP compared with the total ion intensity, respectively (Figure 4a). The Au@TiO_2_ NSs were compared with various previously reported nanomaterials, including Au NPs, MXene, GO, and MoS_2_ sheets [26]. The SY value of the Au@TiO_2_ NSs was estimated to be 63.03 ± 1.64%, which was higher than that of other nanomaterials such as Au NPs (53.82 ± 2.08%), MXene (37.62 ± 2.00%), GO (40.02 ± 2.79%), and MoS_2_ sheets (34.06 ± 2.52%). Furthermore, the DE value of the Au@TiO_2_ NSs was also considerable at 22,720 ± 608. Although it was lower than the DE of GO (30,227 ± 1276), it was higher than those of Au NPs (18,284 ± 4927), MXene (18,527 ± 1503), and MoS_2_ sheets (20,515 ± 3577) (Figure 4b,c). Therefore, the LDI behavior of BP suggested that Au@TiO_2_ NSs can be a more efficient LDI-TOF-MS platform than the other conventional nanomaterials. Especially, the high LDI efficiency of Au@TiO_2_ NSs can be attributed to the star-like morphology of the Au nanocore and the TiO_2_ shell, which leads to an enhanced localized surface plasmon resonance (LSPR) heating effect [27] and thermal desorption accompanied with multiple electronic transitions, respectively [28]. Although it is difficult to clearly determine the dominant factor for the higher LDI efficiency of Au@TiO_2_ NSs than that of Au NPs, the TiO_2_ shell can substantially contribute to the LDI efficiency of Au@TiO_2_ NSs considering its intrinsic LDI property [29].

**Figure 5:**
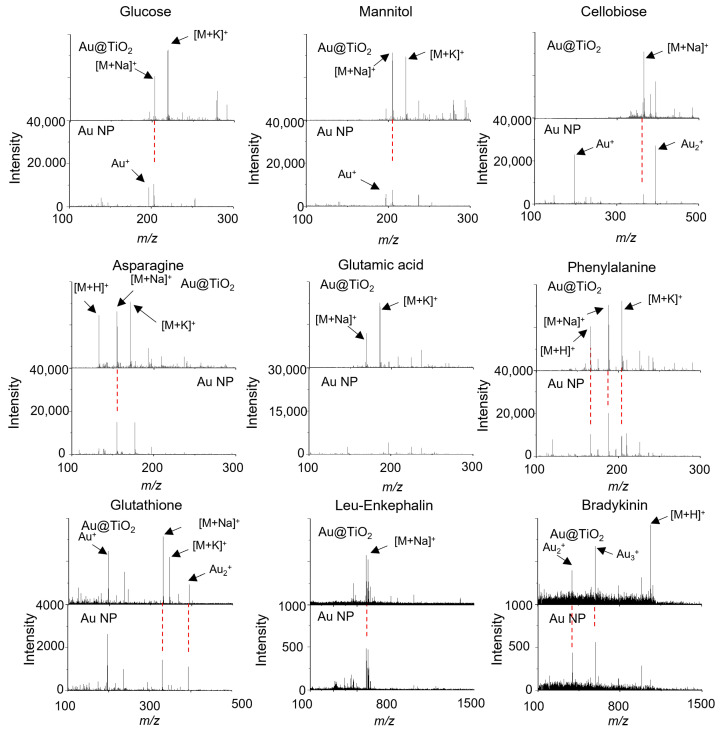
LDI-TOF-MS spectra of 50 pmol glucose, mannitol, cellobiose, Asn, Glu, Phe, glutathione, leu-enkephalin, and bradykinin obtained with Au@TiO_2_ NSs and Au NPs. The red dotted lines indicate the same *m/z* signals.

An LDI-TOF-MS analysis using Au@TiO_2_ NSs was conducted to evaluate its performance with various small molecules, including saccharides, amino acids, and peptides (Figure 5). The LDI efficiency was compared with that of Au NPs to clearly show the effect of the morphology and TiO_2_ shell of the Au@TiO_2_ NSs. All of these analytes were successfully detected with Au@TiO_2_ NSs as the cation adducts with proton, sodium, or potassium, such as *m*/*z* 203 [glucose + Na]^+^, 219 [glucose + K]^+^, 205 [mannitol + Na]^+^, 221 [mannitol + K]^+^, 365 [sucrose + Na]^+^, 381 [sucrose + K]^+^, and 365 [cellobiose + Na]^+^ in saccharides. For amino acids and peptides, the signals were observed at *m*/*z* 133 [Asn + H]^+^, 155 [Asn + Na]^+^, 171 [Asn + K]^+^, 169 [Glu + Na]^+^, 185 [Glu + K]^+^, 156 [His + H]^+^, 178 [His + Na]^+^, 194 [His + K]^+^, 166 [Phe + H]^+^, 188 [Phe + Na]^+^, 204 [Phe + K]^+^, 330 [glutathione + Na]^+^, 346 [glutathione + K]^+^, 579 [Leu-enkephalin + Na]^+^, 595 [Leu-enkephalin + K]^+^, and 1061 [bradykinin + H]^+^, respectively (Figure 5 and Figure S4). The formation of sodium and potassium cation adducts is frequently observed in both MALDI-TOF-MS and LDI-TOF-MS analyses owing to residual impurities of synthesis or incompletely deionized water [30,31]. When the Au@TiO_2_ NSs themselves were analyzed by LDI-TOF-MS, Au cluster ions were strongly observed (Figure S5), but these Au cluster ion signals were suppressed or diminished during the LDI-TOF-MS analysis of small molecules. By stark contrast, the LDI-TOF-MS spectra of small molecules obtained with Au NPs exhibited strong Au cluster ion signals with relatively weak ion intensities of the analytes [32], and there were no signals of the analytes in the cases of Glu and bradykinin (Figure 5 and Figure S4). These results demonstrate that the TiO_2_ shell of Au@TiO_2_ NSs can not only enhance UV absorption capacity but can also effectively suppress the generation of Au cluster ions, prevent energy dissipation through undesired process, and thus facilitate LDI of the analytes. In other words, the TiO_2_ shell plays an important and advantageous role in LDI-TOF-MS analysis through enhancing the LDI efficiency based on its intrinsic optochemical property [33], making the mass signals in LDI-TOF-MS spectra clear without Au cluster ions.

**Figure 6:**
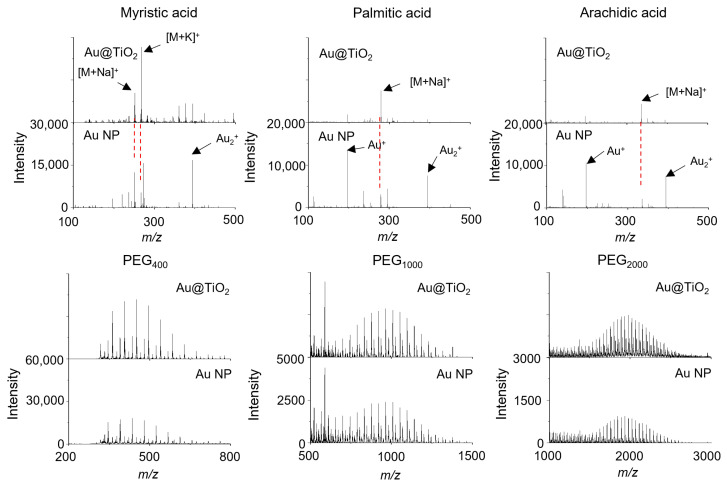
LDI-TOF-MS spectra of 50 pmol myristic acid, palmitic acid, arachidic acid, and PEGs in various molecular ranges obtained with Au@TiO_2_ NSs and Au NPs. The red dotted lines indicate the same *m/z* signals.

The Au@TiO_2_ NSs and Au NPs were also used for the LDI-TOF-MS analysis of myristic acid, palmitic acid, stearic acid, arachidic acid, PEG_400_, PEG_1000_, PEG_2000_, benzo[a]pyrene, and coronene, which are representatives of fatty acids, synthetic polymers, and polyaromatic hydrocarbons (Figure 6 and Figure S4). Most analytes were detected using both Au@TiO_2_ NSs and Au NPs, but benzo[a]pyrene was not detected with Au NPs. Also, the Au cluster ions from the Au NPs were stronger than those from the Au@TiO_2_ NSs, especially in the LDI-TOF-MS spectra of fatty acids (Figure 6). The mass spectrometric signals of the cationized analytes were observed at *m*/*z* 251 [myristic acid + Na]^+^, 267 [myristic acid + K]^+^, 279 [palmitic acid + Na]^+^, 307 [stearic acid + Na]^+^, 335 [arachidic acid + Na]^+^, 253 [benzo[a]pyrene + H]^+^, and 301 [coronene + H]^+^, respectively (Figure 6 and Figure S4). The LDI-TOF-MS spectra of synthetic polymers exhibited a characteristic Gaussian distribution, with intervals of 58 or 44 Da corresponding to their repeating units (Figure 6) [34].

The salt tolerance of the LDI-TOF-MS platform in complex aqueous media is important for further analytical applications such as environmental pollutant and biomarker detection. Therefore, the LDI-TOF-MS performance of Au@TiO_2_ NSs and Au NPs was evaluated with saccharides, amino acids, and fatty acids in a 1X phosphate-buffered saline (PBS) solution (Figure S6). Although numerous unassignable peaks from the PBS were observed in the region below 200 Da, the sodium cation adduct peaks were successfully detected for all analytes including glucose, mannitol, sucrose, cellobiose, Asn, Glu, His, Phe, myristic acid, palmitic acid, stearic acid, and arachidic acid. The Au@TiO_2_ NSs platform exhibited high intensity in the LDI-TOF-MS spectra of most analytes compared with those obtained with Au NPs (Figure S6).

**Figure 7:**
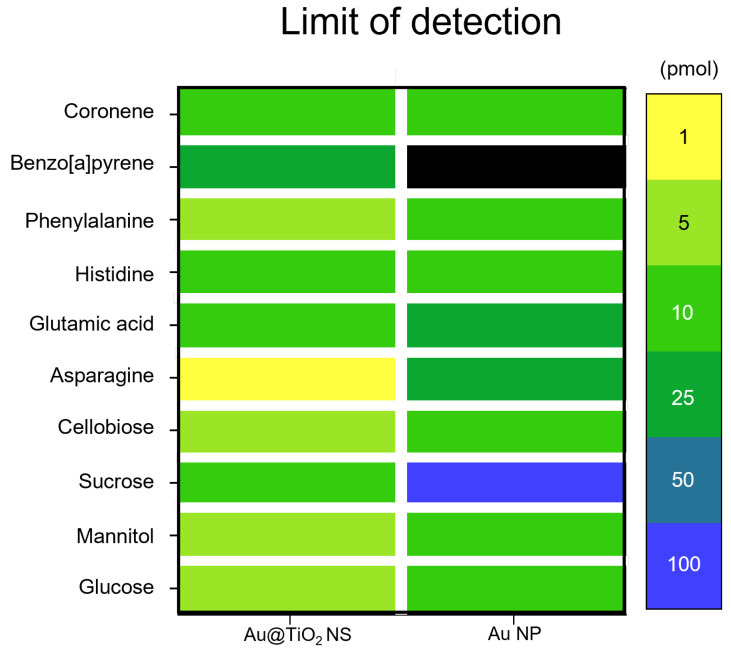
Heat map for limit of detection (LOD) of small molecules obtained with Au@TiO_2_ NSs and Au NPs.

The limit-of-detection (LOD) values for glucose, mannitol, sucrose, cellobiose, Asn, Glu, His, Phe, B[a]P, and coronene were examined with Au@TiO_2_ NSs and Au NPs. The Au@TiO_2_ NSs exhibited LOD values of 5, 5, 10, 5, 1, 10, 10, 5, 25, and 10 pmol, respectively (Figure 7 and Figure S7), indicating superior LDI sensitivity compared with Au NPs, which showed LOD values of 10, 10, 100, 10, 25, 25, 10, 10, 1000, and 10 pmol, respectively (Figure 7).

To assess the quantification capability of Au@TiO_2_ NSs, a dynamic range analysis was also conducted for glucose, mannitol, sucrose, cellobiose, Asn, Glu, His, Phe, B[a]P, and coronene. The dynamic ranges were based on the intensity of sodium cation adduct [M + Na]^+^ peaks for each small molecule. All the analytes showed high linearity in their dynamic ranges, with R-squared values for glucose, mannitol, sucrose, cellobiose, Asn, Glu, His, Phe, B[a]P, and coronene recorded at 0.94792, 0.94087, 0.93621, 0.99721, 0.99382, 0.99306, 0.90325, 0.99111, 0.87031, and 0.92657, respectively (Figure S8). In contrast, when using Au NPs, the R-squared values for glucose, mannitol, cellobiose, Asn, Glu, His, Phe, and coronene were 0.94772, 0.80621, 0.71218, 0.99581, 0.92167, 0.91882, 0.95220, and 0.95650, respectively (Figure S9). The LOD values for sucrose and B[a]P were even too high to establish a dynamic range. Other analytes had a lower signal intensity than that achieved with Au@TiO_2_ NSs. These results suggest that Au@TiO_2_ NSs are more efficient LDI-MS platforms than Au NPs for the quantitative analysis of small molecules.

## 4. Conclusions

Au@TiO_2_ NSs were successfully synthesized via a simple one-pot reaction, and the synthesized Au@TiO_2_ NSs were systematically characterized with various microscopic and spectroscopy tools to reveal their optochemical properties. The Au@TiO_2_ NSs exhibited a strong UV absorption, especially at 343 nm corresponding to the laser wavelength used for LDI-TOF-MS analysis, based on the optochemical interaction between the star-like Au core and the TiO_2_ shell. This interesting optical property of Au@TiO_2_ NSs makes them efficiently harness the laser energy and thus promote the LDI process of the various analytes. Therefore, Au@TiO_2_ NSs showed enhanced SY and DE compared with Au NPs, and additionally, the core@shell structure of Au@TiO_2_ NSs effectively suppressed interference signals from Au clusters themselves, offering improved LODs and salt tolerance with regard to Au NPs. These results suggest that Au@TiO_2_ NSs can serve as a promising and novel matrix for the LDI-TOF-MS analysis of small molecules as well as synthetic polymers owing to their simple synthetic process, high sensitivity, low interference, and salt tolerance.

## Data Availability

Data are contained within the article and Supplementary Materials.

## Supplementary Materials

The following supporting information can be downloaded at https://www.mdpi.com/article/10.3390/nano14231946/s1: Figure S1: DLS data for Au@TiO_2_ NSs; Figure S2: TEM images and UV-Vis spectrum of Au NPs; Figure S3: LDI-TOF-MS spectra of various concentrations of leu-enkephalin, glucose, and His with Au@TiO_2_ NSs; Figure S4: LDI-TOF-MS spectra of 50 pmol sucrose, hidtidine, stearic acid, B[a]P, and coronene obtained with Au@TiO_2_ NSs and Au NPs. Figure S5: LDI-TOF-MS spectrum of Au@TiO2 NSs; Figure S6: LDI-TOF-MS spectra of 50 pmol saccharides, amino acids, and fatty acids in 1X PBS obtained with Au@TiO_2_ NSs and Au NPs; Figure S7: LDI-TOF-MS spectra of various concentrations of saccharides, amino acids, and polyaromatic hydrocarbons obtained with Au@TiO_2_ NSs; Figure S8: Dynamic ranges of saccharides, amino acids, and polyaromatic hydrocarbons obtained with Au@TiO_2_ NSs; Figure S9: Dynamic ranges of saccharides, coronene, and amino acids obtained with Au NPs.
