# Supplementary material for: Laser Desorption/Ionization on Au@TiO2 Core@Shell Nanostars for Mass Spectrometric Analysis of Small Molecules
Source: Nanomaterials (Basel). 2024 Dec 4;14(23):1946. doi: 10.3390/nano14231946 (PMC11643665; doi:10.3390/nano14231946)
Supplement: Supplementary file 1 [file nanomaterials-14-01946-s001.zip › nanomaterials-3337576-supplementary.pdf]

Supporting Information

Laser desorption/ionization on Au@TiO_2 core@shell nanostars for mass spectrometric analysis of small molecules

Hye Sun Cho[1], Jueun Koh[2], Gyeonghye Yim,[2] Hongje Jang[2*] and Young-Kwan Kim[1*]

[1.]Department of Chemistry, Dongguk University-Seoul campus, 30 Pildong-ro, Jung-gu, Seoul 04620, Republic of Korea.

[2.] Department of Chemistry, Kwangwoon University, 20 Gwangwoon-ro, Nowon-gu, Seoul 01897, Republic of Korea.

* To whom correspondence should be addressed.

Correspondence: YKKim: kimyk@dongguk.edu; HJang: hjang@kw.ac.kr

Supporting Figures

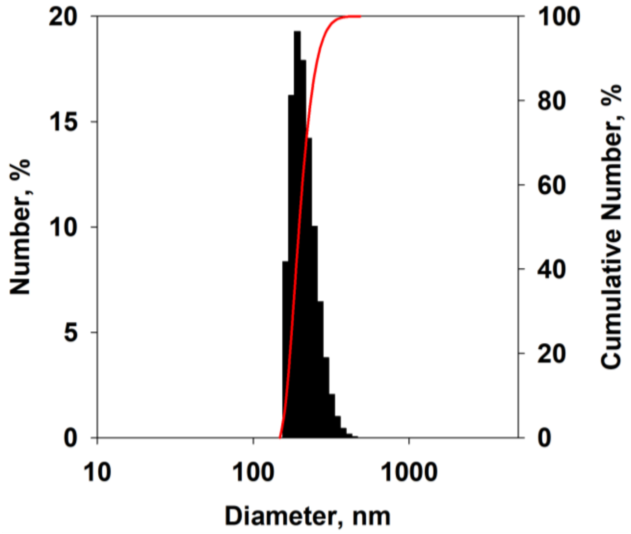

Figure S1. DLS data for Au@TiO_2 NSs.

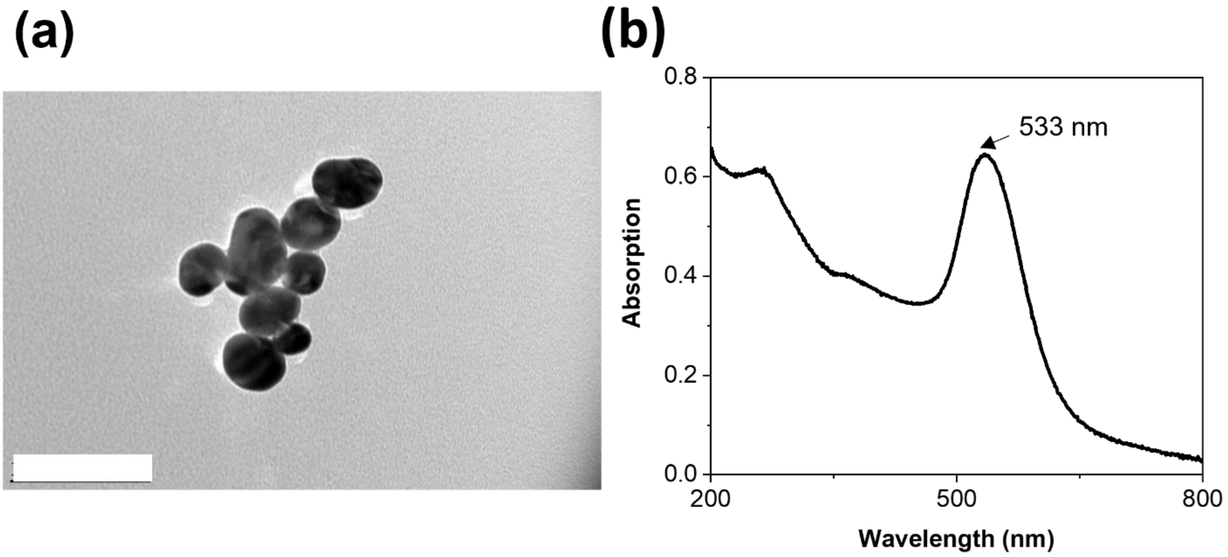

Figure S2. (a) TEM images and (b) UV-Vis spectrum of Au NPs. The scale bar is 100 nm.

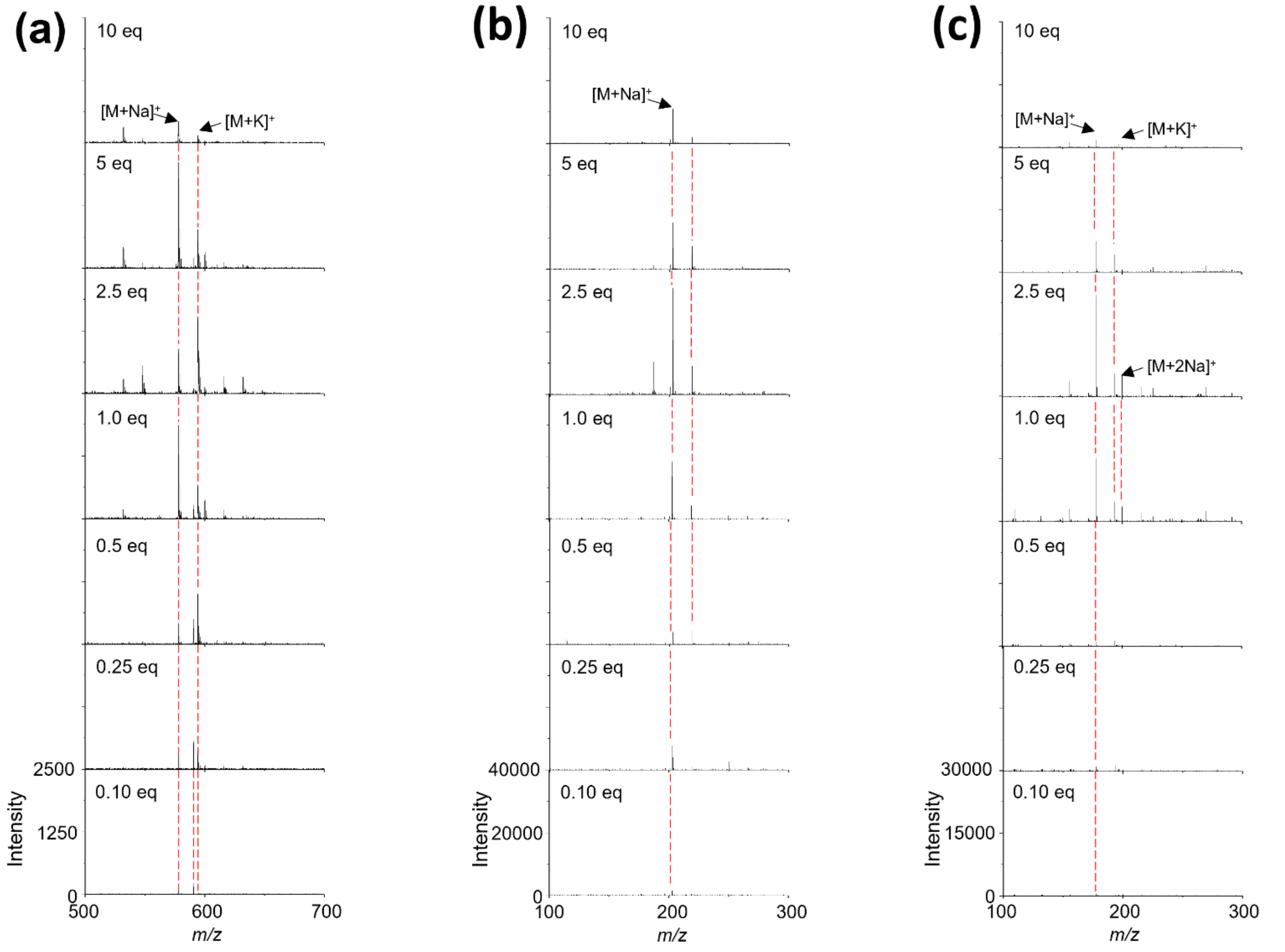

Figure S3. Concentration optimization of Au@TiO_2 NSs as LDI-TOF-MS platform. LDI-TOF-MS spectra of various concentrations of (a) Leu-enkephalin, (b) glucose and (c) His with Au@TiO_2 NSs.

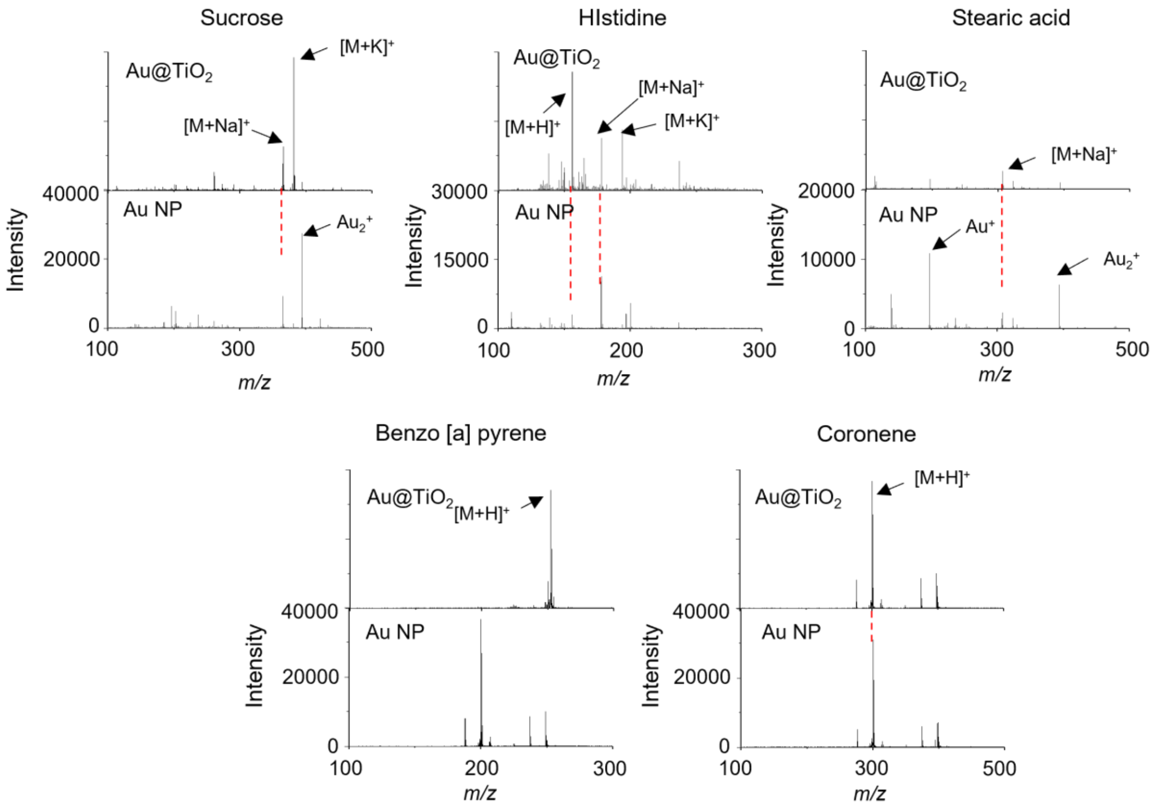

Figure S4. LDI-TOF-MS spectra of 50 pmol sucrose, histidine, stearic acid, B[a]P, and coronene obtained with Au@TiO_2 NSs and Au NPs.

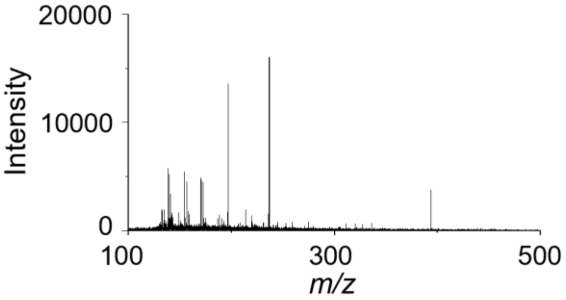

Figure S5. LDI-TOF-MS spectrum of Au@TiO_2 NSs.

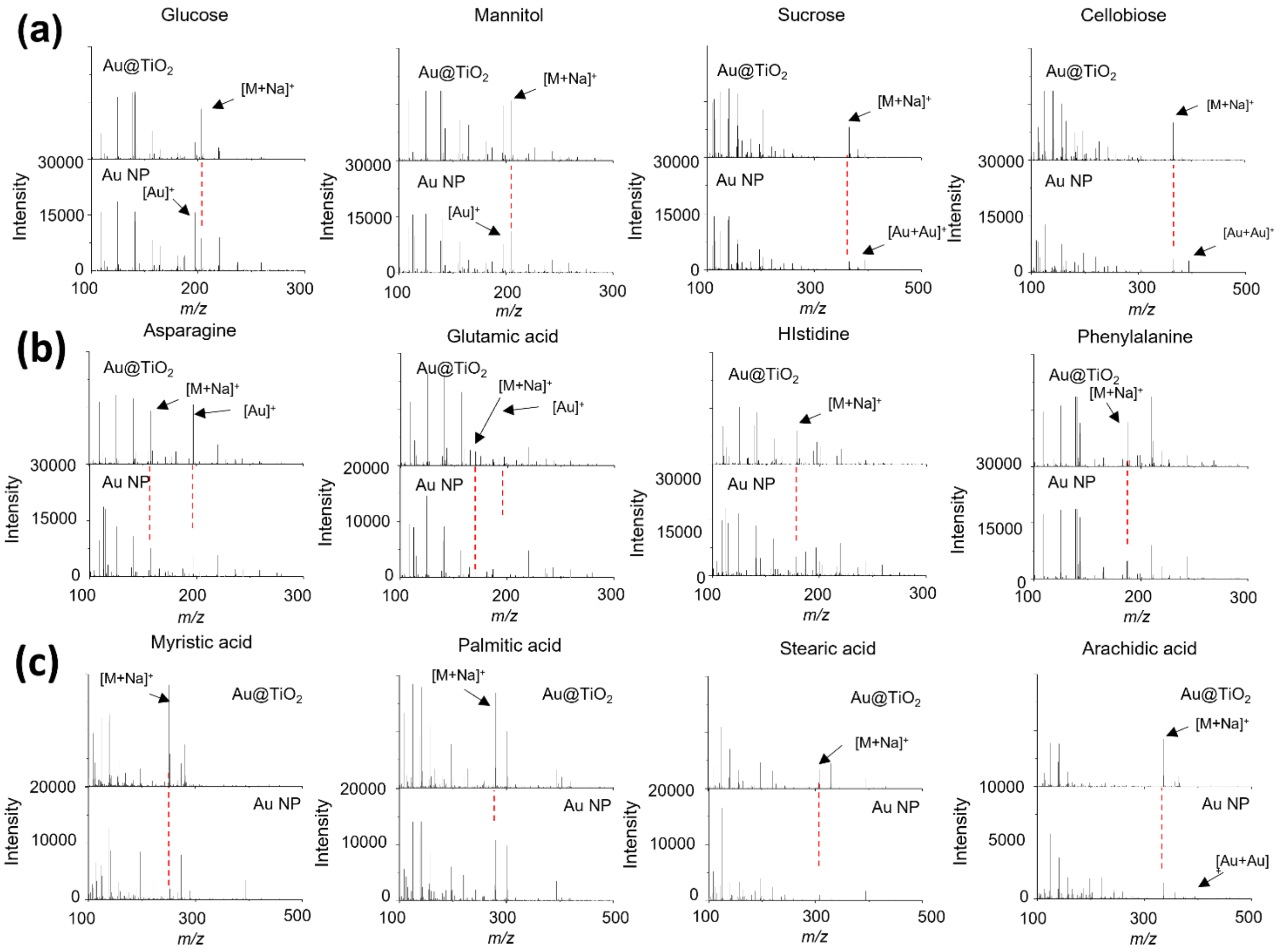

Figure S6. LDI-TOF-MS spectra of 50 pmol (a) saccharides, (b) amino acids, and (c) fatty acids in 1X PBS solution obtained with Au@TiO_2 NSs and Au NPs.

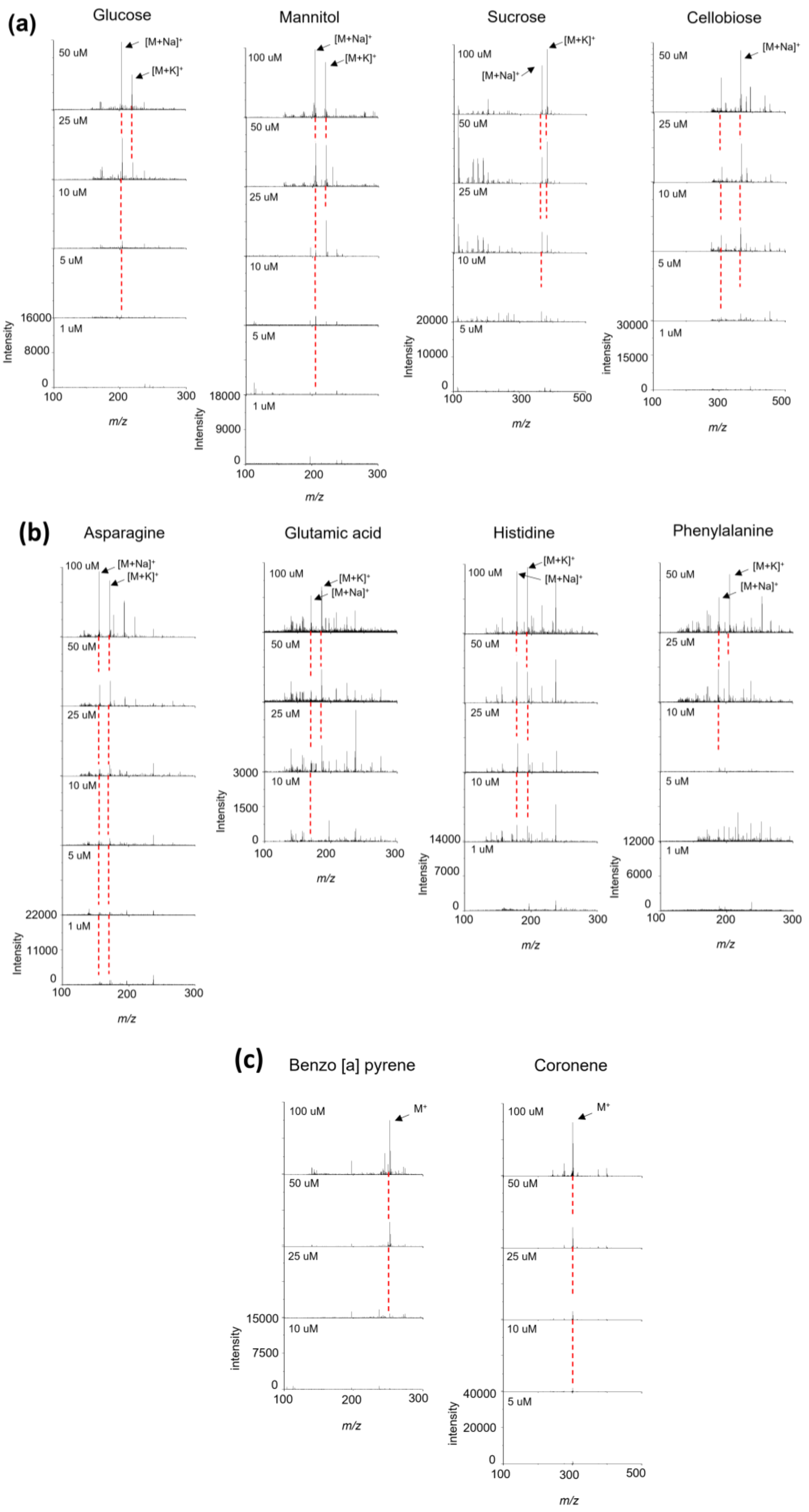

Figure S7. LDI-TOF-MS spectra of various concentrations of (a) saccharides, (b) amino acids and (c) polyaromatic hydrocarbons obtained with Au@TiO_2 NSs.

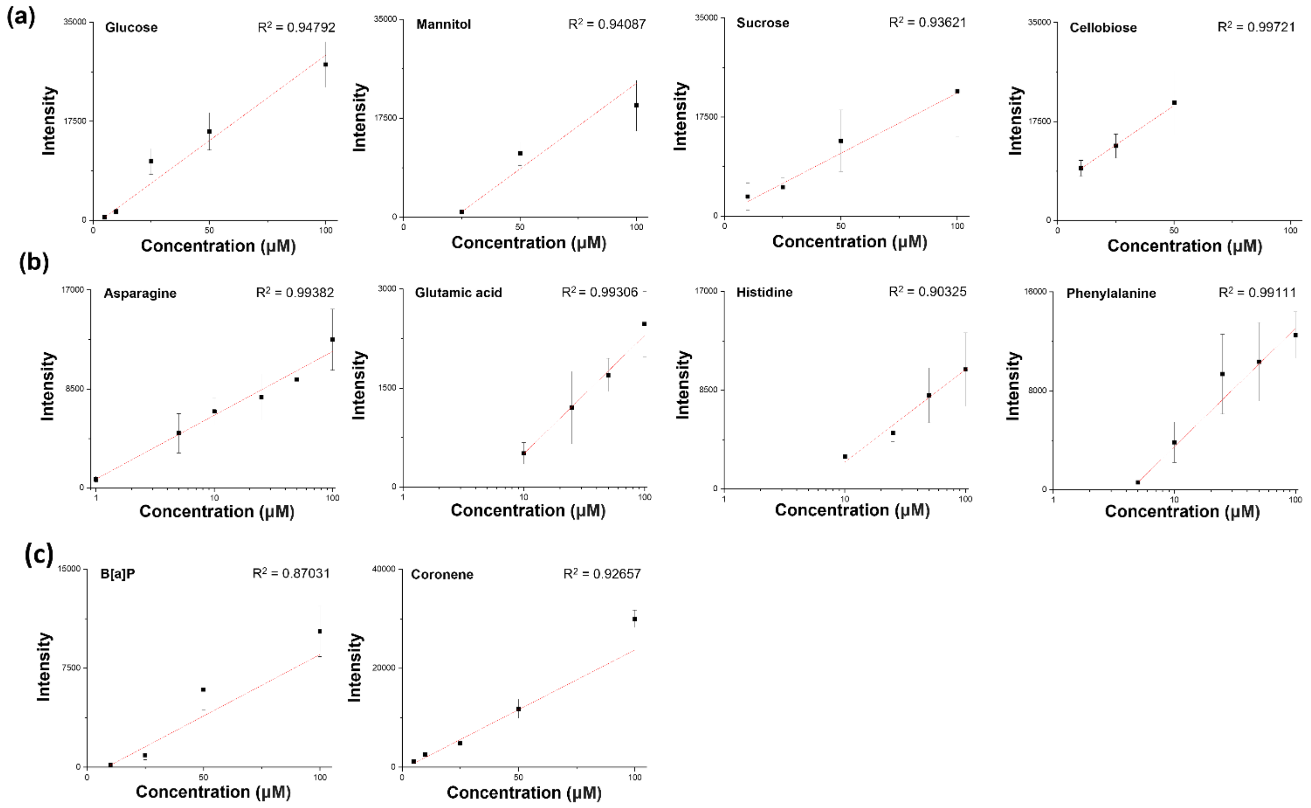

Figure S8. Dynamic ranges of (a) saccharides, (b) amino acids, and (c) polyaromatic hydrocarbons obtained with Au@TiO_2 NSs.

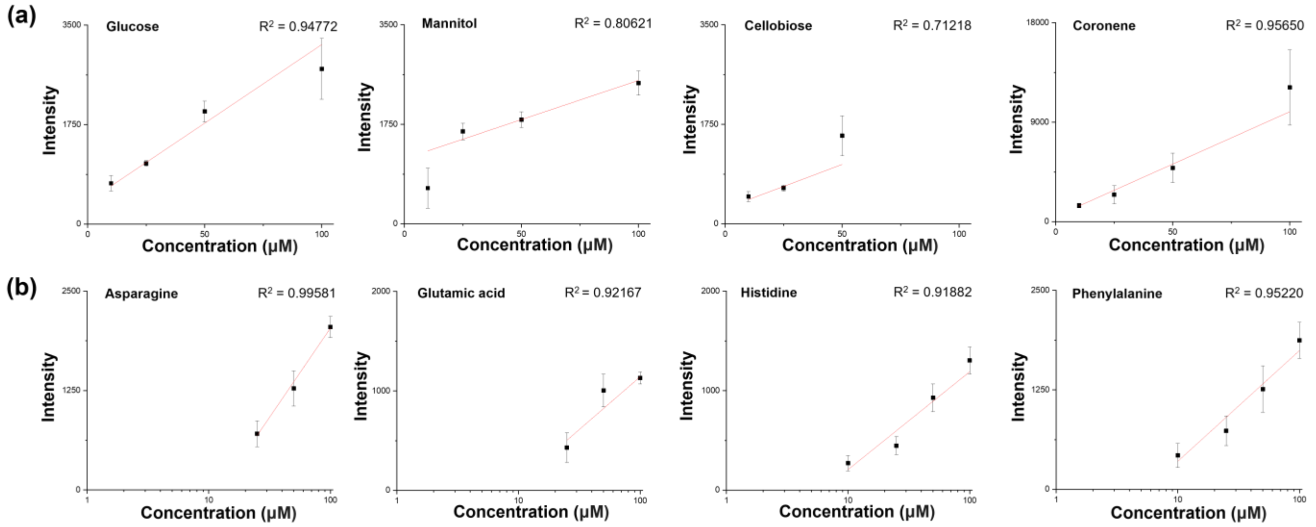

Figure S9. Dynamic ranges of (a) saccharides, coronene, and (b) amino acids obtained with Au NPs.
